# Glycemic Variability: An Independent Predictor of Mortality and the Impact of Age in Pediatric Intensive Care Unit

**DOI:** 10.3389/fped.2020.00403

**Published:** 2020-07-31

**Authors:** Yuhui Du, Chengjun Liu, Jing Li, Hongxing Dang, Fang Zhou, Yuelin Sun, Feng Xu

**Affiliations:** ^1^Department of Medicine Intensive Care Unit, Children's Hospital Affiliated to Zhengzhou University, Henan Children's Hospital, Zhengzhou Children's Hospital, Zhengzhou, China; ^2^Department of Pediatric Intensive Care Unit, Children's Hospital of Chongqing Medical University, Ministry of Education Key Laboratory of Child Development and Disorders, National Clinical Research Center for Child Health and Disorders, China International Science and Technology Cooperation Base of Child Development and Critical Disorders, Chongqing Key Laboratory of Pediatrics, Chongqing, China

**Keywords:** glycemic variability, pediatrics, critical care, mortality, age

## Abstract

**Objective:** To compare the ability of different indices of glycemic variability (GV) in the prognostic evaluation of critically ill children and investigate whether heterogeneity of glucose control exists within this population group.

**Methods:** We conducted a retrospective study of the GV data collected from patients admitted to the pediatric intensive care unit, Children's Hospital of Chongqing Medical University between January 2016 and December 2016. We calculated the mean glucose level (MGL) and four indices of GV, namely, standard deviation (SD), coefficient of variation (CV), mean amplitude of glycemic excursion (MAGE), and glycemic lability index (GLI). The 28-day mortality was considered as the primary endpoint.

**Results:** Survivors and non-survivors showed significant differences in terms of the SD, CV, MAGE, and GLI (*P* < 0.05, for all). However, GLI was superior to the other indices and showed an independent association with ICU mortality (odds ratio [OR], 1.082; 95% confidence interval [CI], 1.031–1.135; *P* < 0.01). Sub-group analysis disaggregated by quartiles of MGL and GV revealed that younger subjects (age ≤ 36 months) had significantly higher mortality in the lowest quartile of the MGL and in the highest quartile of GV; the older children (age > 36 months) experienced significantly higher mortality in the highest quartiles of MGL and GV.

**Conclusion:** GV is closely associated with mortality, and among all glucose parameters evaluated, GLI was found to be the strongest predictor of outcomes. This paper is the first report of age being a potentially important modifier of the association between GV, MGL, and mortality in critically ill children.

## Introduction

Dysglycemia is a common complication of severe illnesses. Several researchers have investigated the effects of blood glucose levels on the prognosis of various conditions, but the results of these studies have been conflicting, particularly in regard to whether tight glycemic control (TGC) does indeed improve clinical outcomes as compared to standard care (STD) protocols (1–6). One possible explanation for this discrepancy is increased glycemic variability (GV), which is believed to augment the risk of hypoglycemia as well as the overall mortality (7).

Studies have shown an independent relationship between GV and mortality in heterogeneous populations of critically ill patients (8–19), a population group in which GV has a stronger association with mortality than hyperglycemia and hypoglycemia (8). Generally, accepted guidelines also recommend that GV be reported in studies that investigate the management of blood glucose (BG) levels (20). Therefore, the study of GV is crucial to the development of an effective glucose control protocol.

GV is known to increase the risk of mortality and affect the functioning of multiple organs (9, 21, 22), especially the development of the nervous system in children (23). However, no guidelines or relevant literatures have yet been published on GV and fluctuation range in critically ill children. At present, data from pediatric studies on GV and outcomes are limited, and GV is assessed mainly on the basis of the index that lower and higher than the therapeutic range (9, 21, 22). However, if the index does not take into account other glucose values, the time sequence of measurement, and intervals between measurements, it may not be the most accurate parameter to assess GV. Compared with the number of studies on GV in adults, studies on critically ill children are limited. The following are the four indices of GV that are most often calculated: standard deviation (SD), coefficient of variation (CV), mean amplitude of glycemic excursion (MAGE), and glycemic lability index (GLI). These indices are used to predict the risk of mortality (11, 17). Currently, no guidelines have been established on the best index for the evaluation of GV. In recent years, GLI has been adopted as the preferred index of GV in adults to integrate both the range and rapidity of glucose fluctuation.

However, it is yet to be determined whether the findings of adult studies are applicable to critically ill children. Therefore, this study was designed to determine whether there is an association between the risk of mortality and the SD, CV, MAGE, and GLI, which are used to assess GV; this study also sought to identify the best indicator of GV. Previous studies have shown that when children have a tight glycemic control, their insulin requirements vary by age, and a 60-day age cut off is recommended for differential infection rate (24, 25). Hence, we aim to further study whether there is a heterogeneous effect of GV in different age groups.

## Materials and Methods

### Study Design and Setting

The retrospective study was conducted at a 31-bed pediatric intensive care unit (PICU), which is a combined unit for both medical and surgical patients at the Children's Hospital of Chongqing Medical University, Chongqing, China. The children were classified on the basis of whether they were survivors or non-survivors, with respect to the primary endpoint is 28-day mortality after PICU admission. Long-term follow-up was performed by PICU nurses one month after discharge by telephonic or interview (the follow-up questionnaire is shown in the annex).

### Inclusion and Exclusion Criteria

The patients included consecutive children admitted to the PICU between January 2016 and December 2016; the age of the patients ranged from >36 weeks (corrected gestational age) and <16 years old, and only children who had stayed in the PICU for at least 3 day were enrolled. Patients who had a clinical diagnosis of diabetes mellitus or inherited metabolic diseases and those who have <2 BG measurements per day were excluded from this study. For children with multiple PICU admissions, only the last admission was taken into consideration for this study.

### Clinical and Laboratory Data

Data regarding the following parameters were collected from the records of the clinical and laboratory variables: general demographic data, 24-h PRISM III score, 72-h glucose levels, treatment details (72-h renal-replacement therapy, use of insulin infusion, or steroid therapy, need for 24-h invasive mechanical ventilation [IMV]), length of stay in the PICU (PICU LoS), length of stay in hospital (hospital LoS), the number of survivors, and mortality within 28 days of PICU admission.

### Management of Blood Glucose Level

Arterial blood gas analysis was performed routinely for all patients since the day of admission to the ICU. To ensure homogeneity of glucose measurements, we only used the BG measurements obtained from arterial blood samples, at a minimum interval of 12 h. During the time period at which the data for this study were collected, there was no standardized practice for the monitoring of blood glucose, and the frequency of BG monitoring was adjusted according to the patient's condition. Therefore, the glucose measurements for all the enrolled patients had been made at the discretion of the attending physicians.

The mean glucose level (MGL) was calculated according to all the available BG values. The four indices of GV were calculated: SD, CV, and MAGE were defined as the mean of absolute values of any glucose (consecutive values) readings that are >1 SD of all the BG values (11, 17). GLI was determined using the following formula:

$$\begin{array}{l} \text{GLI}\left[{\left(\text{mmol}/\text{L}\right)}^{2}/\text{h}/\text{d}\right]=\left[\sum \left(\Delta \text{B}{\text{G}}^{2}/\Delta \text{h}\right)\right]/\text{d}, \end{array}$$

where ΔBG, difference between glucose concentration; Δh, time interval (the minimum time interval was 1 h and the maximum time intervals was 12 h), d, number of days of BG monitoring (11, 17).

### Statistical Analysis

All statistical analyses were performed using the SPSS version 19.0 (SPSS Inc.). We compared the variables related to the clinical outcome using the Chi-squared test or Mann–Whitney *U*-test, as appropriate. The data are presented as mean ± SD or as median and interquartile range (IQR), depending on the distribution of the variables. The correlation between the parameters related to GV and PRISM III scores was assessed using Spearman correlation analysis. Receiver operating characteristic (ROC) curve analysis was performed to compare the different parameters in terms of their ability to predict ICU mortality. Logistic regression models were established to identify independent predictors of mortality. Furthermore, patients were categorized into three subgroups on the basis of age, and further assigned to sub-categories using quartiles of MGL and GV (quartiles 1, 2+3, and 4). *P*-values of <0.05 were considered indicative of statistical significance.

## Results

**Figure 1:**
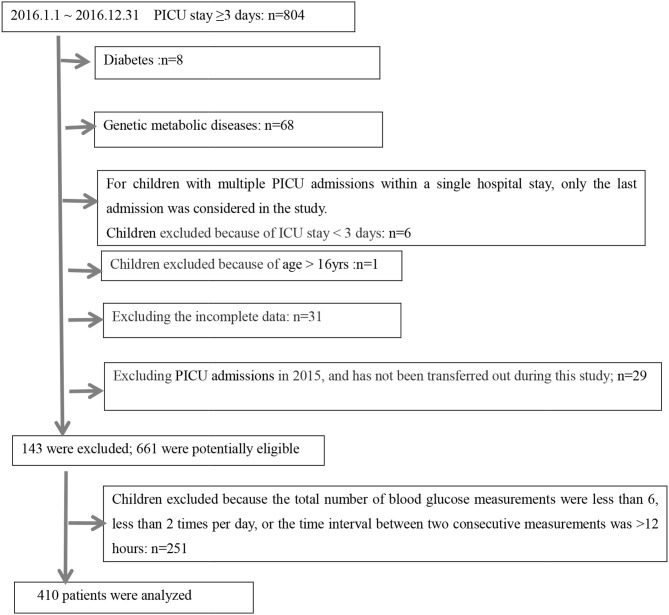
Schematic illustration of the study population and patient selection criteria.

A schematic illustration of the protocol for eligibility in this study is shown in Figure 1. In all, 410 critically ill children were enrolled in this study, 84 (20.5%) of them died during their stay in the PICU. The clinical features of the survivor group and non-survivor group were compared (Table 1); a total of 4,242 BG measurements were included in the analysis. The diagnosis at the time of admission included respiratory diseases (30.5%), neurological diseases (16.1%), sepsis (3.7%), cardiac surgery (18.3%), thoracoabdominal surgery (13.4%), and others (18.0%). Both groups differed significantly in terms of admission diagnosis, PRISM III score, need for IMV, and steroid therapy (*P* < 0.05, for all). All GV indices in the non-survivor group were significantly higher than those in the survivor group (*P* < 0.05). However, we also observed that the ICU LoS was shorter for survivors than for non-survivors, but the differences in the LoS between the two groups were not significant (*P* > 0.05). In contrast, the hospital LoS was significantly longer for the survivors than for non-survivors (*P* < 0.001). The need for insulin infusion was determined by the attending physician. Since only three critically ill children were administered insulin infusion during their ICU stay, this variable could not be analyzed further.

**Table 1:** Comparison between characteristics of non-survivors and survivors.

**Characteristic**	**Total (*n* = 410)**	**Survivors (*n* = 326)**	**Non-survivors (*n* = 84)**	**χ^2^/*Z***	***P*-value**
Age (months)				1.348	0.246
0–36	308 (75.1)	249 (76.4)	59 (70.2)		
>36	102 (24.9)	77 (23.6)	25 (29.8)		
Sex, (Male, %)	239 (58.3)	194 (59.5)	45 (53.6)	0.969	0.325
Weight (kg)	8.0 (4.5, 15.0)	7.5 (4.5, 15.0)	9.0 (5.0, 16.0)	−0.845	0.398
Admission diagnosis				18.240	0.003
Respiratory	125.0 (30.5)	98.0 (30.1)	27.0 (32.1)		
Neurological	66.0 (16.1)	47.0 (14.4)	19.0 (22.6)		
CV surgery	75.0 (18.3)	72.0 (22.1)	3.0 (3.6)		
Surgical (non-CV)	55.0 (13.4)	44.0 (13.5)	11.0 (13.1)		
Sepsis	15.0 (3.7)	12.0 (3.7)	3.0 (3.6)		
Other	74.0 (18.0)	53 (16.3)	21 (25.0)		
IMV	351 (85.6)	271 (83.1)	80 (95.2)	7.950	0.005
Renal-replacement	51 (12.4)	42 (12.9)	9.0 (10.7)	0.289	0.591
Steroid	168 (41.0)	149 (45.7)	19 (22.6)	14.719	0.000
PRISM III score	9.0 (5.0, 14.0)	8.0 (5.0, 13.0)	11.0 (7.3, 18.0)	−4.601	0.000
PICU Los/day	7.0 (5.0, 11.0)	7.0 (5.0, 11.0)	8.0 (5.0, 13.8)	−0.922	0.357
Hospital Los/day	21.0 (13.0, 30.0)	23.0 (15.0, 31.0)	11.0 (6.0, 20.8)	−6.865	0.000
MGL, mmol/L	5.9 (5.3, 6.8)	5.9 (5.3, 6.7)	6.1 (5.3, 7.3)	−1.378	0.168
SD, mmol/L	1.4 (1.0, 2.0)	1.4 (1.0, 2.0)	1.6 (1.2, 2.3)	−2.868	0.004
CV	0.2 (0.2, 0.3)	0.2 (0.2, 0.3)	0.3 (0.2, 0.3)	−2.792	0.005
MAGE, mmol/L	2.5 (1.7, 3.9)	2.4 (1.7, 3.8)	2.9 (1.9, 4.4)	−2.058	0.040
GLI, (mmol/L)2/h/d	1.5 (0.5, 4.0)	1.4 (0.4, 3.5)	2.5 (0.8, 5.5)	−3.552	0.000

*Data presented as median (IQR) or as frequency (%). CV surgery, cardiovascular surgery; IMV, invasive mechanical ventilation; PRISM III, Pediatric Risk of Mortality III; Los, length of stay; MGL, mean glucose level; CV, coefficient of variation; MAGE, mean amplitude of glycaemic excursion; GLI, glycaemic lability index*.

### Correlation Analysis

All the four GV indices investigated in this study (SD, CV, MAGE, and GLI) as well as MGL showed a significant association with the PRISM III score (*r* = 0.284, 0.246, 0.265, 0.404, and 0.218, respectively; *P* < 0.001, for all). On the basis of the correlation coefficient, a strong correlation was observed between GLI and PRISMIII score.

### Receiver Operating Characteristic Curve Analysis

**Figure 2:**
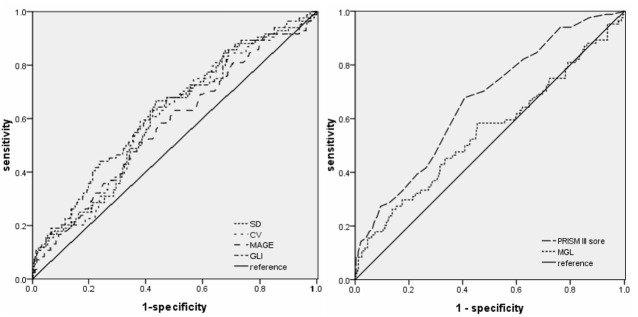
Receiver operating characteristic curves of GLI, SD, CV, MAGE, MGL, and PRISM III score for ICU death in the entire cohort (*n* = 410).

Receiver operating characteristic (ROC) curves were plotted to evaluate the effectiveness of GV indices, PRISM-III score, and MGL in predicting ICU mortality (Figure 2). The area under the ROC curves were as follows: 0.626 (95% confidence interval [CI]: 0.558–0.693) for GLI; 0.601 for (95% CI: 0.535–0.668) SD; 0.599 (95% CI: 0.531–0.666) for CV; 0.573 (95% CI: 0.503–0.642) for MAGE; 0.549 (95% CI: 0.475–0.623) for MGL, and 0.662 (95% CI: 0.599–0.726) for PRISM III score. These results show that GLI is superior to other GV indices in the prediction of ICU mortality and is the closest to the PRISM III score. Further, MGL did not show any discriminative ability for ICU mortality.

### Logistic Regression Analysis for Predictors of Mortality

Correlation analysis revealed that among the four indices of GV studied, GLI showed the strongest association with PRISM-III score and had the highest discriminative value (Figure 2). Therefore, GLI was used as the index of GV in logistic regression analysis (Table 2). The dependent variable was ICU mortality, whereas the independent variables were GLI, MGL, age, category of diagnosis at admission, PRISM III score, need for IMV, and steroid therapy. Because PRISM III scores include the glucose level, collinearity diagnostics was performed before logistic regression analysis. Variance inflation factor (VIF) and tolerance values of GV were 1.323 and 0.756, respectively, while those of PRISM-III were 1.231 and 0.812; these values indicate the absence of significant collinearity between PRISM III scores and glucose level. Thus, we found that among the variables taken into consideration, GLI, PRISM-III score, need for IMV, and steroid therapy bore an independent correlation with mortality (*P* < 0.05), whereas MGL and other variables did not show such a correlation.

**Table 2:** Results of logistic regression analysis showing predictors of mortality in critically ill children.

**Variables**	**Parameters**	**Wald χ^2^**	***P*-value**	**OR (95% CI)**
Age ≤ 36 m	>36 m	0.025	0.875	0.943 (0.452–1.964)
Diag	Respiratory	20.885	0.001	
Surgical (non-CV)		5.287	0.021	0.342 (0.137–0.853)
CV surgery		15.114	0.000	0.055 (0.013–0.237)
Sepsis		0.540	0.462	0.568 (0.126–2.566)
Neurological		1.497	0.221	0.563 (0.225–1.413)
Other		0.081	0.777	1.133 (0.478–2.684)
No-IMV	IMV	12.550	0.000	0.102 (0.029–0.361)
No-steroid	steroid	4.276	0.039	2.187 (1.042–4.593)
PRISM III score		18.291	0.000	1.106 (1.056–1.158)
MGL, mmol/L		1.141	0.286	1.148 (0.891–1.477)
GLI, (mmol/L)2/h/d		10.140	0.001	1.082 (1.031–1.135)

*CV, cardiovascular surgery; IMV, invasive mechanical ventilation; PRISM III, Pediatric Risk of Mortality III; MGL, mean glucose level; GLI, glycaemic lability index*.

### Association of GV With Unfavorable Outcome Based on Mean Glucose Level

When MGL and GV were categorized into quartiles, patients in the highest GV quartile had the highest mortality, both in the overall study population and in the different age groups. Among all patients, only the subgroup with the lowest MGL showed a significant increase in mortality with an increase in GV (*P* < 0.05); in addition, mortality was the highest for the subgroup with lowest MGL and highest GV. A similar tendency was observed in the case of younger subjects (age ≤ 36 months). However, among patients in the older age group (age > 36 months), mortality was the highest for the subgroup with the highest percentile of MGL and highest percentile of GV, although the differences between the subgroups of the different quartiles were not statistically significant.

## Discussion

In this retrospective study, we investigated the correlations between GV, mortality, and age in critically ill children. All the evaluated indices of GV, namely, SD, CV, MAGE, and GLI, showed a direct association with unfavorable clinical outcomes in critically ill children. Among the four indices, GLI showed the strongest association with mortality and was found to be superior to other parameters in reflecting GV. One of the key aspects of this study was that we also conducted sub-group analysis, which revealed a potential modifier—age—had a significant impact on the clinical outcome. In the younger age group (age ≤ 36 months), mortality was the highest for those in the lowest quartile of MGL and the highest quartile of GV. In the older subjects (age > 36 months), mortality was the highest among patients in the highest quartiles of both MGL and GV, although the differences between the subgroups of different quartiles were not statistically significant.

In our study, we noted that survivors had shorter ICU Los but significantly longer hospital LoS as compared to non-survivors. This discrepancy may be explained by the fact that the condition of the survivors may be relatively less severe than that of the non-survivors, whereby the survivors could be transferred out of the PICU earlier, resulting in a shorter ICU stay. Further, most of the non-survivors had died in the PICU, since their condition was worse than survivors. Therefore, non-survivors had longer ICU LoS and relatively shorter hospital LoS as compared to survivors.

Recent studies have suggested that increased oxidative stress may play an important role in the GV-induced worsening of the clinical outcomes in ICU patients. Studies have shown that GV has a more detrimental effect than chronic sustained hyperglycemia (26) and that it is associated with multiple-organ dysfunction (21, 22), particularly neurologic impairment (23, 27). Univariate analysis in this study revealed that the studied values of the GV-related indices were significantly higher in the non-survivors than in the survivors (*P* < 0.05). However, multivariate logistic regression analysis revealed that GV is independently associated with ICU mortality, even after controlling for MGL and disease severity. These findings are consistent with those of recent retrospective studies that have shown that GV is a predictor of mortality among adult and pediatric ICU patients (8–19). Put together, these findings indicate that GV indices can be used for the prognostic assessment of critically ill ICU patients.

Studies on the association between GV and mortality have already been conducted in diverse populations of ICU patients. However, the best index of GV is yet to be identified. A study in China has shown that PRISM-III score is a robust indicator of prognosis in critically ill children (28). Our study revealed that GLI has the strongest correlation with PRISM-III score as compared to all the other BG parameters taken into consideration in this study. On ROC curve analysis, GLI was found to have better ability to predict ICU mortality as compared to SD, CV, and MAGE. These findings are similar to those reported previously. Donati et al. (11) reported that area under the curve (AUC) for GLI, SD, CV, and MAGE is 0.62, 0.60, 0.60, and 0.59, respectively. Zuo et al. (12) reported that the AUC for GLI was 0.64 and that for MGL was 0.56. Ali et al. (17) reported that the AUC for GLI, SD, and MAGE was 0.67, 0.62, and 0.59, respectively. One explanation for the consistently superior performance of GLI across different studies may be that GLI completely reflects the GV by integrating the range, speed, time series, and time interval between BG measurements. On the other hand, both SD and CV do not account for the successive changes in measurements and their timing (11, 13). The value of MAGE depends on the frequency of sampling and outlier BG values tend to disproportionately affect the value of MAGE (11). Thus, GLI is a superior parameter for truly reflecting GV. The PRISM-III score has the drawbacks of focusing only on the impact of hyperglycemia on mortality, without taking into account the effect of hypoglycemia; GV summarizes the effect of both hyperglycemia and hypoglycemia in critically ill patients. Therefore, by using GLI to evaluate GV, the prognosis of critically ill children can be better evaluated.

**Figure 3:**
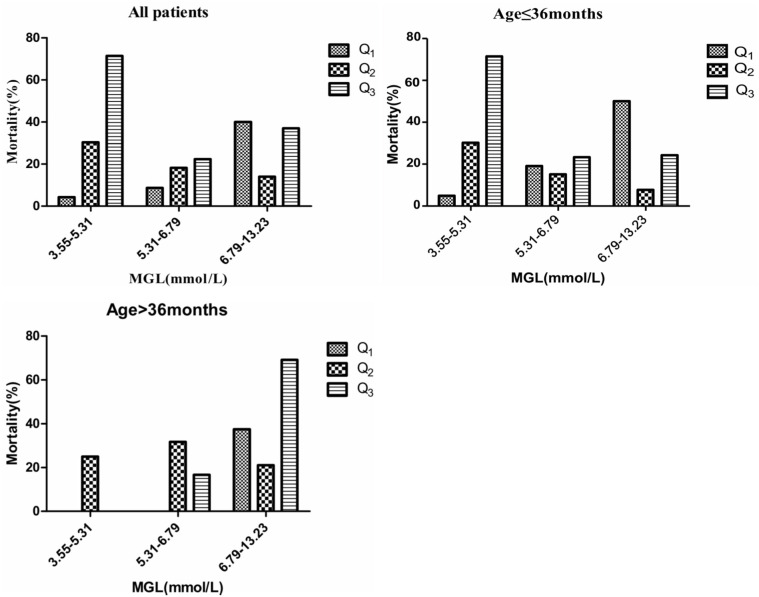
Mortality in different quartiles of MGL. GV calculated by GLI. *P*-values denote differences in mortality across GV categories between MGL, GV, and mortality.

Our findings have shown that GV is associated with mortality. GLI was found to be a better predictor of clinical outcomes as compared to SD, CV, or MAGE. Several studies in adults have shown that GV influences the clinical outcome in medical and surgical patients admitted to the ICU. Donati et al. (11) have shown that on categorizing patients according to GLI quartile and further stratifying by MGL, patients in the upper GV category and the highest quartile of MGL exhibited the highest adjusted odds ratio of poor prognosis. Both Farrokhi et al. (19) and Okazaki et al. (29) have also reported similar results. These findings imply that higher values of mean glucose level and GV may be associated with worse prognosis. Similar to the methods used in the abovementioned studies, we classified blood glucose levels into quintiles on the basis of MGL to evaluate the association between GV and outcomes (Table 3 and Figure 3). The findings for older children (age > 36 months) appeared to be similar to those reported previously for adults. Among the younger subjects (age ≤ 36 months), those with the lowest percentile of MGL and highest GV showed the highest mortality rate. To the best of our knowledge, no studies hitherto have identified an age cut-off for differences in the relation between the MGL, GV, and mortality. This age-related discrepancy in the relation between MGL, GV, and mortality may be attributed to pathophysiological differences; poor storage ability and high metabolism of liver glycogen in younger children may render them more vulnerable to hypoglycemia than older children and adults (9, 30). Younger children are known to be more susceptible to the adverse effects of hypoglycemia as compared to their older counterparts, and hypoglycemic episodes are know to be associated with an increased risk of mortality (31). The age of the patient has been identified as an important modifier in some studies on glycemic control and outcomes. Agus et al. (25) have shown that that compared to older patients, younger patients have higher rate of infections. Another observational cohort study has shown that the hyperglycemia induced incremental risk in mortality in neonates is less than that in older children (32). When tight glycemic control is targeted in children, insulin may be required at significantly low doses in younger children after adjusting for body mass index (on multivariate analysis, body mass index is not associated with cumulative insulin requirement) (24). Therefore, it is reasonable to conclude that the effect of GV on mortality is heterogeneous across the study population. Therefore, glucose control strategies in clinical settings should account for age-related variation in clinical response.

**Table 3:** Mortality in various subgroups disaggregated by quartiles of MGL and GV.

**MGL quartiles (mmol/L)**	**GLI quartiles (mmol/L)**^****2****^**/h/day, Mortality**, ***n*****/*****N*** **(%)**			**χ^2^**	***P*-value**
	**Q_**1**_**	**Q_**2+3**_**	**Q_**4**_**		
**All patients (*****n*** **=** **410)**					
Quartile A_1_, (3.55–5.31)	2/46 (4.3)	14/46 (30.4)	5/7 (71.4)	20.757	0.000
Quartile A_2_+A_3_, (5.31–6.79)	4/46 (8.7)	20/110 (18.2)	11/49 (22.4)	3.633	0.304
Quartile A_4_, (6.79–13.23)	4/10 (40.0)	7/50 (14.0)	17/46 (37.0)	7.546	0.056
**Age** **≤** **36 months (*****n*** **=** **308)**					
Quartile A_1_, (3.55–5.31)	2/41 (4.9)	13/43 (30.2)	5/7 (71.4)	18.856	0.000
Quartile A_2_+A_3_, (5.31–6.79)	4/21 (19.1)	14/92 (15.2)	10/43 (23.3)	1.764	0.623
Quartile A_4_, (6.79–13.23)	1/2 (50.0)	2/26 (7.69)	8/33 (24.2)	4.291	0.179
**Age** **>** **36 months (*****n*** **=** **102)**					
Quartile A_1_, (3.55–5.31)	0/6 (0.0)	1/4 (25.0)	0/0 (0.0)	2.960	0.400
Quartile A_2_+A_3_, (5.31–6.79)	0/24 (0.0)	7/22 (31.8)	1/6 (16.7)	10.676	0.006
Quartile A_4_, (6.79–13.23)	3/8 (37.5)	4/19 (21.1)	9/13 (69.2)	7.065	0.065

This study has some merits. First, this is the first study that evaluates the association between GV (showed by SD, CV, MAGE, and GLI) and mortality in a diverse population of critically ill children admitted to the PICU. Previous studies on GV in critically ill children have employed only a single index of GV (8–10, 21–23). One of the key findings of our study is the differential effect of GV on the outcomes in the age groups of ≤ 36 and > 36 months.

Our study also has some limitations. This is a single-center, retrospective cohort study; therefore, it was not possible to rule out the effect of several confounding factors on our findings. For example, ICU patients may often require nutritional support, which might affect glucose homeostasis; however, the guidelines for nutritional assessment recommend a delay in parenteral nutrition, and withholding parenteral nutrition for 1 week has been associated with better prognosis as compared to earlier parenteral nutrition, in critically ill children (33, 34). The BG levels of the children were monitored for 72 h; therefore, nutritional status was not included in our analysis. In this study, only three critically ill children were administered insulin infusion during ICU stay, and we do not account for the effects of insulin in these cases. Additionally, other potential confounders, such as the use of drugs and intravenous glucose, could not be evaluated. However, our sample size is large enough, and the study has adequate power to testify the association between association GV and mortality. Since this was a retrospective study, there were also some limitations regarding the lack of standardization of glucose intake and frequency or timing of glucose measurements, which may influence the estimation of GV. However, the use of continuous BG monitoring will help us overcome these limitations.

## Conclusions

Our study demonstrates that GV is independently associated with ICU mortality and that compared to the other investigated indices of GV, GLI is a better predictor of mortality in critically ill children. Furthermore, we found that the relationship between GV our findings also revealed a differential effect between patients aged ≤ 36 months and those aged >36 months. We recommend that glucose control in younger people should be carefully assessed with special attention to the central tendency, variability, and minimum value, while central tendency, variability, and maximum value merit more attention in older patients. The results of this exploratory analysis are meant to inform subsequent researches on blood sugar in critically ill children, and we need more prospective experiments to acquire a better target glucose range for critically ill children in different ages.

## Data Availability Statement

All datasets generated for this study are included in the article/Supplementary Material.

## Ethics Statement

The studies involving human participants were reviewed and approved by the ethical committee of Children's Hospital of Chongqing Medical University (File No. 2019, 37).

## Author Contributions

JL contributed to the conception of the study. YD and CL contributed significantly to analysis and manuscript preparation and performed the data analyses and wrote the manuscript. HD, FZ, YS, and FX helped perform the analysis with constructive discussions. All authors contributed to the article and approved the submitted version.

## Conflict of Interest

The authors declare that the research was conducted in the absence of any commercial or financial relationships that could be construed as a potential conflict of interest.
