# Supplementary material for: Glycemic Variability: An Independent Predictor of Mortality and the Impact of Age in Pediatric Intensive Care Unit
Source: Front Pediatr. 2020 Jul 31;8:403. doi: 10.3389/fped.2020.00403 (PMC7412867; doi:10.3389/fped.2020.00403)
Supplement: Supplementary file 1 [file Data_Sheet_1.docx]

**the follow-up forms of PICU**

1.The current condition of the children who have been discharged【[comment](javascript:;)】

○

○ Dead children, not involved

2. the condition after discharged【[comment](javascript:;)】

○ Not treatment

○ Rehabilitation in our hospital

○ Continue treatment at the local hospital

○ Continue treatment in a better hospital

○ Dead children, not involved

3. What else help is needed now【[comment](javascript:;)】

○Basic nursing

○Medication guidance

○ Rehabilitation at home

○Psychological help

○Fertility guidance

○Medical insurance reimbursement

○The [inspection](javascript:;) results

○Needn't any help

4. Satisfaction with our work, comments and Suggestions【[comment](javascript:;)】

○[Great](javascript:;) [satisfaction](javascript:;)

○[Satisfaction](javascript:;)

○General satisfaction

○Dissatisfaction

| [confirm](javascript:;) |
| --- |

| eliminate |
| --- |

**2**. **table 4:collinearity diagnostics**

| model | Unstandardized Coefficients | | Standardized Coefficients | t | Sig | Collinearity statistics | |
| --- | --- | --- | --- | --- | --- | --- | --- |
|  | B | standard error |  |  |  | tolerance | VIF |
| constant | .840 | .143 |  | 5.880 | .000 |  | |
| Diag | .002 | .012 | .011 | .194 | .846 | .670 | 1.492 |
| Age | .043 | .050 | .047 | .862 | .389 | .724 | 1.381 |
| IMV | -.192 | .058 | -.167 | -3.308 | .001 | .823 | 1.215 |
| steroid | .184 | .042 | .225 | 4.438 | .000 | .822 | 1.217 |
| PRISM III score | .010 | .003 | .162 | 3.188 | .002 | .812 | 1.231 |
| MGL | .015 | .017 | .050 | .860 | .390 | .632 | 1.581 |
| GV | .012 | .004 | .171 | 3.233 | .001 | .756 | 1.323 |
| variable: prognosis | | | | | | | |
